# SOCIAL NETWORK SIZE AND THE TRANSITION TO NONDRIVING

**DOI:** 10.1093/geroni/igad104.0621

**Published:** 2023-12-21

**Authors:** Kellia Hansmann, Ronald Gangnon, Carolyn McAndrews, Stephanie Robert

**Affiliations:** William S Middleton Memorial Veterans Hospital, Madison, Wisconsin, United States; University of Wisconsin-Madison, Madison, Wisconsin, United States; University of Wisconsin Madison, Madison, Wisconsin, United States; University of Wisconsin-Madison, Madison, Wisconsin, United States

## Abstract

Having family and friends available to provide rides is an important coping factor after people stop driving. Social networks may also be a means by which older adults can reduce or stop driving earlier while maintaining their mobility. We examined whether social network size was associated with 3-year changes in driving behavior among U.S. older adults. We analyzed data on community-dwelling drivers in the National Health and Aging Trends Study (n = 5,140; 2015 cohort). We categorized whether participants reported zero, one, or two or more people to talk to about important things (who lived in their county). We used weighted logistic regression models to estimate whether having more same-county people to talk to about important things was associated with incidence of any of four new driving avoidance behaviors (avoiding driving: at night, in bad weather, on highways, or alone) or driving cessation, at three-year follow up, adjusting for biopsychosocial variables. There was no significant association between having two or more same-county people to talk to about important things and the odds of driving cessation at follow up (adjusted Odds Ratio [aOR]: 1.00, 95% Confidence Interval [CI]: 0.58-1.71) compared to drivers who reported having no one to talk to about important things. Similarly, there was no significant association with the incidence of one or more new driving avoidance behaviors (aOR: 1.16 95% 95% CI: 0.91-1.47). Future research should examine whether other aspects of social networks (e.g., type, quality, closer proximity) might play important roles in supporting the transition to non-driving.

